# First reported case of *Pascalia glauca* poisoning in a pediatric patient: a case report

**DOI:** 10.3389/fmed.2025.1589190

**Published:** 2025-09-10

**Authors:** Andrea Milena Rodríguez-Guerrero, Tania Galán, Marjorie Rodríguez-Guerrero, César Patiño-Rocha, Jorge Vasconez-Gonzalez, Esteban Ortiz-Prado

**Affiliations:** ^1^Ministry of Public Health, Atacames Health Center, Esmeraldas, Ecuador; ^2^Pediatrics Department, Vicente Corral Moscoso Hospital, Cuenca, Ecuador; ^3^Intensive Care Department, Vicente Corral Moscoso Hospital, Cuenca, Ecuador; ^4^One Health Research Group, Faculty of Health Sciences, Universidad de Las Américas, Quito, Ecuador

**Keywords:** *Pascalia glauca*, *Wedelia glauca*, carboxyatractyloside, plant toxicity, neurotoxicity, hepatotoxicity, acute liver failure, pediatric poisoning

## Abstract

**Background:**

*Pascalia glauca* is a toxic plant species predominant in South America, known to cause severe liver and kidney damage, and even death, in animal studies. Its toxicity is attributed to carboxyatractyloside, a compound that inhibits the transport of adenosine triphosphate (ATP) into the mitochondria, leading to cellular dysfunction. To date, no cases of human poisoning have been reported.

**Case report:**

We present the case of a 6-year-old male who developed tonic-clonic seizures, altered consciousness, vomiting, and a Glasgow Coma Scale score below 8 after ingesting *Pascalia glauca*. A cranial CT scan revealed small intraparenchymal petechial hemorrhages in the biparietal and frontal regions. The patient was started on anticonvulsant and neuroprotective treatment with levetiracetam. Laboratory findings showed markedly elevated transaminases and coagulopathy, which did not improve with vitamin K administration, leading to a diagnosis of acute liver failure. The patient was treated with rifaximin, N-acetylcysteine, and lactulose, showing progressive improvement in liver function. After 27 days in the pediatric intensive care unit (ICU), he was transferred to the general ward hemodynamically stable but with persistent neurological impairment, unable to speak or respond to stimuli.

**Conclusion:**

This first reported case of human poisoning by *Pascalia glauca* highlights the plant’s potential neurotoxic and hepatotoxic effects in humans. The absence of prior human cases poses challenges in management and prognosis, emphasizing the need for increased awareness among healthcare professionals and further research on its toxicological impact.

## 1 Introduction

*Pascalia glauca*, formerly classified as *Wedelia glauca*, is a toxic plant commonly known as sunchillo or clavel amarillo. It belongs to the Asteraceae family and is widely distributed across Argentina and neighboring South American countries, including Bolivia, Brazil, Chile, Paraguay, and Uruguay (1). However, its presence has not been documented in Venezuela, Colombia, or Peru. The introduction of this plant into Ecuador is suspected to have occurred through trade and agricultural exchanges with countries where it is endemic (2).

Several species within the *Asteraceae* family are known to be toxic to humans and domestic animals (3). *Pascalia glauca* is a perennial herbaceous plant that thrives in various soil types, reaching a height of 30–80 cm. It has rigid stems, lanceolate leaves with serrated edges, and yellow terminal capitulum flowers (3).

The toxicity of *Pascalia glauca* is attributed to carboxyatractyloside, a diterpenoid glycoside concentrated in its seeds and newly formed cotyledons. This compound acts as a competitive inhibitor of the adenine nucleotide transporter (ANT) in mitochondria, disrupting oxidative phosphorylation and blocking adenosine triphosphate (ATP) transport (4). The primary organ affected is the liver, where intoxication leads to diffuse hemorrhagic necrosis and acute liver failure, characterized by centrolobular necrosis with hemorrhagic infiltration (5).

While *Pascalia glauca* toxicity has been extensively documented in livestock, no cases in humans have been reported until now. Intoxication in ruminants (cattle and sheep) leads to severe hepatic necrosis, with post-mortem findings including swollen livers with a “nutmeg liver” appearance, as well as neurological and respiratory distress (6). A recent outbreak of spontaneous *Pascalia glauca* poisoning in sheep in Argentina affected 20% of a commercial flock, leading to ataxia, mucous nasal discharge, cough, and respiratory distress (6). Experimental studies have confirmed lethal outcomes in goats, with death occurring within 9–24 h post-ingestion due to irreversible liver failure (5). The toxic potential of this plant extends beyond fresh consumption; dried plant material in hay has also been associated with fatal outcomes in cattle (7).

## 2 Case presentation

**FIGURE 1:**
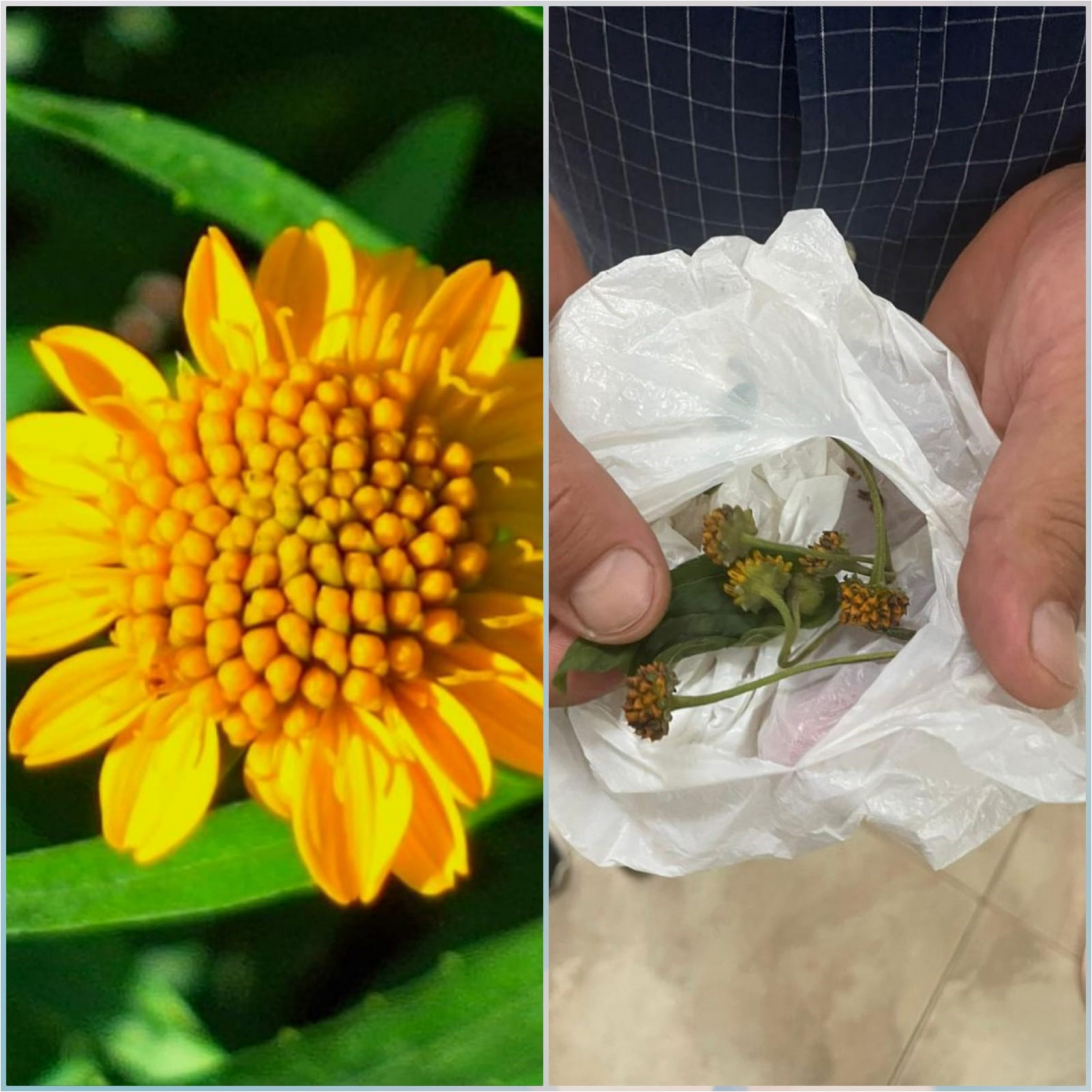
*Pascalia glauca*.

We present the case of a 6-year-old male patient from Morona Santiago province, Eastern Ecuador, with an incomplete vaccination schedule. The patient was referred 24 h after the accidental ingestion of a plant of initially unknown origin. Following interviews with family members, healthcare personnel, and local community members, the plant was identified as *Pascalia glauca* (Figure 1).

The first symptoms appeared 6 h post-ingestion, consisting of multiple tonic-clonic seizures lasting approximately 2 min each, with no postictal recovery. Due to geographic access limitations, the patient first sought medical attention 10 h after ingestion at a local healthcare facility.

Upon arrival at the hospital, the patient exhibited progressive neurological deterioration, with impaired consciousness, repeated episodes of vomiting, and a Glasgow Coma Scale (GCS) score of <8, indicating severe encephalopathy. Given his critical condition, endotracheal intubation was performed, and basic supportive measures were initiated. Laboratory tests revealed hypoglycemia during the initial evaluation.

## 3 Diagnostic evaluation

Upon admission, the patient underwent comprehensive laboratory testing, revealing significant abnormalities. The hematologic panel showed leukocytosis with a white blood cell count of 34.5 × 10^9^/L, predominantly neutrophilia (30,040/mm^3^) with a reduced lymphocyte percentage (5.6%), while the platelet count remained within normal limits at 452,000/mm^3^. Hemoglobin levels were slightly decreased (10.8 g/dL), suggesting mild anemia. The coagulation profile indicated a prolonged prothrombin time (PT) of 23.8 s with reduced PT activity (36%), an elevated international normalized ratio (INR) of 2.02, and a prolonged activated partial thromboplastin time (aPTT) of 51.4 s (Table 1), all consistent with coagulopathy. An increase in procalcitonin levels was observed in the patient, which can be explained by pneumonitis that progressed to pneumonia as a result of bronchoaspiration, suspected due to the presence of foul-smelling secretions at the time of endotracheal tube change.

**TABLE 1:** Relevant results during the patient’s evolution.

Admission and follow-up laboratory tests	Results	Reference values
Admission tests	White blood cells: 34.5	5.0–12.0
	Neutrophils: 87.3 (neutrophils: 30,040/mm^3^)	25–43
	Lymphocytes: 5.6	32–61
	Platelets: 452.0	140–450
	PT: 23.8	9.8–13.5
	PT%: 36	70–130
	INR: 2.2	0.9–1.1
	aPTT: 51.4	22–36.9
	Urea	27,3
	Creatinine: 0.48	
	Total bilirubin: 1.8	0–1.1
	Direct bilirubin: 0.7	0–0.3
	AST: 1372	0–40
	ALT: 1167	0–50
Admission arterial blood gas (ABG)	pH: 7.1	7.35–7.45
	pCO2: 48.4	35–45
	pO2: 117.5	
	Lactate: 5.8	0.5–2–20
	HCO3: 12.1	21–29
	Base excess: −17.3	
Liver function and acute phase reactants 24 h	Total bilirubin: 1.7	0–1.1
	Direct bilirubin: 0.8	0–0.3
	AST: 3418	0–40
	ALT: 2291	0–50
	CRP:2.7	0–0.5
	Procalcitonin: 10.7	>0.046
Liver function 12 h after	AST: 4764	0–40
	ALT: 6057	0–50
	Albumin: 3.3	3.8–5.4
	PT: 25.1	9.8–13.5
	PT%: 33	70–130
	INR: 2.14	0.9–1.1
	aPTT: 43.4	22–36.9
Immunological tests	-ANA (antinuclear antibodies): positive -ANCA (anti-neutrophil cytoplasmic antibodies): Negative	
Peripheral blood smear	Anemia pattern: microcytic hypochromic Eosinophils: none Blasts: none	
Renal function alteration (10 days)	Urea: 249	10–50
	Creatinine: 5.5	
	Uric Acid: 13	3.4–7
Improvement of liver function	AST: 56.7	0–40
	ALT: 98.5	0–50
	PT: 11.5	9.8–13.5
	PT%: 112.8	70–130
	INR: 0.96	0.9–1.1
	aPTT: 33.5	22–36.9

Liver function tests were markedly abnormal, with aspartate aminotransferase (AST/TGO) at 1372 U/L and alanine aminotransferase (ALT/TGP) at 1167 U/L, indicating significant hepatocellular injury. Additionally, lactate dehydrogenase (LDH) was elevated at 1164 U/L, suggesting extensive cellular damage. C-reactive protein (CRP) remained low at 0.4 mg/dL, suggesting the absence of systemic inflammation. Renal function tests initially showed normal creatinine levels at 0.48 mg/dL, but mild hyponatremia (134 mEq/L) and hyperkalemia (5.34 mEq/L) were present.

Arterial blood gas analysis demonstrated severe metabolic acidosis, with a pH of 7.10, bicarbonate (HCO3) of 12.1 mmol/L, and a base excess of −17.3, alongside an elevated lactate level of 8.4 mmol/L, suggesting significant anaerobic metabolism likely due to systemic hypoxia and mitochondrial dysfunction. Urine toxicology screening was positive for benzodiazepines, likely secondary to prior sedation or previous medical interventions, and intoxication by amphetamines, barbiturates, cocaine, cannabis, methadone, morphine, phencyclidine, methamphetamines, nortriptyline was excluded.

**FIGURE 2:**
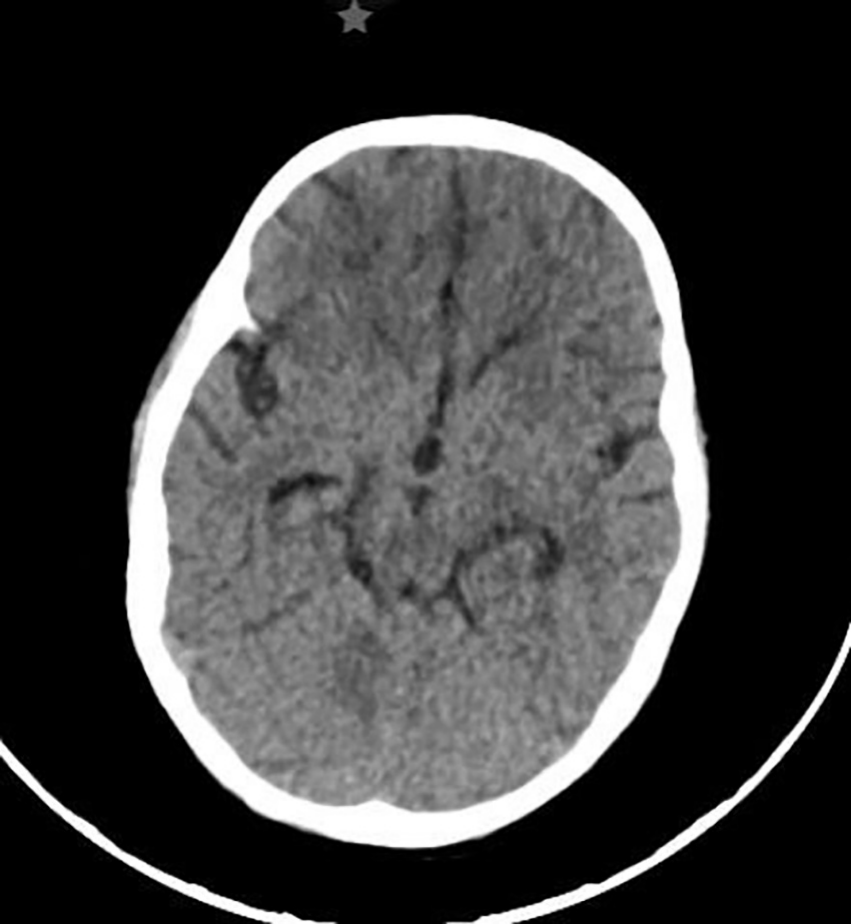
Cranial Computed Tomography (CT) Scan with Evidence of Hematomas. The CT scan revealed hypodense lesions in the frontal region, consistent with bleeding that progressed toward the convexity. There was no displacement of the midline.

During hospitalization, additional diagnostic imaging and neurological assessments were conducted. Chest X-rays taken during the patient’s hospitalization show atelectasis, no signs of pulmonary effusion, and clear costophrenic angles. Abdominal ultrasound revealed a thickened gallbladder wall, likely indicative of reactive changes due to the presence of free intraperitoneal fluid (ascites). Given the patient’s neurological deterioration, an electroencephalogram (EEG) was performed, demonstrating generalized slow-wave activity, consistent with metabolic encephalopathy, likely secondary to hepatic dysfunction and neurotoxicity. Once stabilized, a cranial computed tomography (CT) scan with and without contrast was obtained, revealing small intraparenchymal petechial hemorrhages in the biparietal and frontal regions, with no evidence of midline shift or significant mass effect, no signs of cerebral edema were evident (Figure 2). These findings suggested coagulopathy-associated microvascular injury, likely a result of hepatic failure-induced dysfunction in clotting factor synthesis.

Given the complexity of the patient’s presentation, a multidisciplinary approach was undertaken. Gastroenterology specialists were consulted for management of acute liver failure and coagulopathy, while the neurology team monitored for ongoing seizure activity and metabolic encephalopathy progression. Hematology specialists evaluated coagulation abnormalities and potential transfusion needs, while nephrology was involved due to renal function deterioration and electrolyte imbalances. Additionally, ophthalmology evaluation was requested to assess potential toxic effects on the visual pathways. Given the severity of the patient’s condition, intensive supportive care was initiated in the Pediatric Intensive Care Unit (ICU), with continuous monitoring of hepatic, renal, and neurological status.

## 4 Therapeutic intervention

**-Neurological:** Due to a history of seizures and neurotoxicity described in animals, anticonvulsant and neuroprotective treatment with levetiracetam was started. During hospitalization, the patient was maintained on sedoanalgesia with midazolam, fentanyl, and rocuronium bromide in continuous infusion. Upon detecting altered liver function in laboratory tests, rocuronium bromide was discontinued, and midazolam dosage was reduced due to reports of hepatotoxicity in the literature. Encephalopathy was confirmed through an electroencephalogram. Upon discharge from the Pediatric Intensive Care Unit (PICU), the patient maintained a Glasgow Coma Scale score of 10.

**-Respiratory:** Invasive mechanical ventilation was initiated for 20 days with varying ventilatory parameters during hospitalization, maintaining hypoxemia related to respiratory distress and thoracic restriction caused by anasarca. After starting peritoneal dialysis, consistent improvement was observed, achieving adequate blood gas objectives.

**-Hemodynamic:** Upon arrival, the patient was unstable with low blood pressure for his age. A double inotropic support regimen was started with epinephrine and norepinephrine infusions, resulting in partial control of blood pressure. Milrinone was added to improve perfusion. The patient was considered to be in vasogenic shock refractory to amines, so hydrocortisone was initiated, stabilizing the hemodynamic status. On day 10 of hospitalization, after discontinuing vasoactive drugs, the patient presented blood pressures above the 90th percentile, this can be explained by the patient’s renal failure, associated with withdrawal syndrome due to prolonged use of sedation. Hydralazine was started, but blood pressure control remained poor, requiring nitroprusside, losartan, and amlodipine infusions to maintain adequate blood pressure under strict monitoring by Pediatric Nephrology.

**-Digestive:** On admission, the patient had significantly altered transaminases and coagulation times, which did not correct with vitamin K administration, leading to a diagnosis of acute liver failure. Hepatomegaly 6 cm below the costal margin and ascites were observed. Rifaximin, N-acetylcysteine, and lactulose were initiated as part of the management. The patient was kept NPO (nothing by mouth) and was given a gastric protector, with improvement in liver function. Nutrition via nasogastric tube was gradually introduced with good tolerance, but as the patient did not exhibit adequate swallowing mechanics, a gastrostomy tube was placed for feeding.

**-Renal:** Upon arrival, the patient had adequate renal function. However, in the days following admission, refractory edema and renal injury were noted. Albumin and diuretic support were initiated. Due to acidosis, volume overload, anuria, hyperkalemia, and increased uric acid, peritoneal dialysis was started with successful response. Despite the option of hemodialysis, the patient required peritoneal dialysis because it is a less invasive procedure, it is the one available in our setting. Furthermore, the patient presented a high risk of bleeding with prolonged clotting times, so hemodialysis was not considered the best option.

**-Metabolic:** Upon arrival, the patient presented with metabolic acidosis and underwent repeated bicarbonate corrections. Additionally, the patient experienced hyperkalemia and hypokalemia, requiring multiple internal balance corrections during hospitalization.

**-Hematological:** The patient presented with epistaxis, for which phytomenadione, tranexamic acid, and fresh frozen plasma were administered, leading to improved coagulation times and cessation of bleeding. Eosinophilia was noted in the studies, and the patient remained under hematology surveillance.

**-Infectious:** Triple antibiotic therapy with ceftriaxone, clindamycin, and vancomycin was started due to invasive procedures, skin lesions, suspicion of sepsis, and immunosuppression related to the underlying condition. The patient exhibited poor clinical evolution, with persistent fever and increased acute-phase reactants. Antibiotics were changed to cefotaxime and meropenem. During hospitalization, *Ascaris lumbricoides* was observed exiting through the endotracheal tube, and pyrantel pamoate was added to the treatment regimen.

## 5 Patient’s evolution

The patient remains in the pediatric ICU for 27 days and is transferred to the general ward hemodynamically stable, not uttering words, nor responding to stimuli. In the pediatric clinic ward, feeding is advanced with good tolerance, and antibiotic therapy is discontinued. Physical therapy is carried out. An MRI is performed, showing multiple hyperdensities in the cortex and white matter, probably secondary to cerebral edema, so anticonvulsant therapy is maintained. Discharge is planned with referral for physical and respiratory therapy.

## 6 Discussion

According to reports from various poison control centers in North America and Europe, exposure to plants is one of the most common types of human poisoning, accounting for about 5% of all cases (8). The toxicity of plants is due to a wide variety of chemical toxins, including alkaloids, glycosides, proteins, and amino acids. Toxicity varies depending on the part of the plant and the route of exposure, whether dermal, oral, or inhalation (8, 9). The most affected age group is the pediatric population, which is believed to be due to the accessibility and attractiveness of plants for this age group. In the case of young children, most cases are due to water ingestion, while in the adolescent population, there have been reports of plant consumption for recreational purposes or self-harm (8).

In most cases, adverse effects often do not occur or are generally mild, and no therapeutic intervention is necessary. It is estimated that in around 20% of cases, symptoms are mild to moderate (8, 9). Moderate symptoms include high fever, cold sweating, difficulty swallowing, pain in the oral cavity, nausea, vomiting, diarrhea, headache, paresthesia, peripheral tremor, and balance disorders. Severe symptoms typically include liver and kidney failure, coma, and cardiopulmonary arrest (8, 10, 11). The most common manifestations are gastrointestinal in nature, occurring in 46% of cases, followed by neurological manifestations (33%), mostly anticholinergic effects, and 14.9% suffering from mucosal lesions and corrosions (12).

However, some plants are extremely toxic, and ingesting small amounts can cause rapid death. Such is the case with *Pascalia glauca*. Animal studies (goats) showed that after ingesting this plant, death occurred between 9 and 24 h post-poisoning (5). Animal studies have revealed that the liver can become congested, enlarged, and darker than usual. Histopathological findings include severe centrolobular to midzonal hemorrhagic coagulative necrosis, and it has also been observed that the nuclei of hepatocytes were pyknotic, while other cells showed karyolysis or karyorrhexis, as well as cytoplasmic fragmentation and hyper-eosinophilia (1).

In the patient, a liver biopsy could not be performed due to significant coagulation disturbances and the risk of complications, which prevented confirming the presence of centrolobular hepatic necrosis and hemorrhage described in other studies. Among the findings described in animals that can be extrapolated to our case are liver and kidney damage, seizures, and treatment-refractory coagulopathy. A new finding, yet to be investigated, includes cerebral microhemorrhages, ascitic-edematous syndrome, respiratory distress syndrome, and hypoglycemia.

The diagnosis of plant poisoning can be challenging. In this case, causes of poisoning were ruled out by a urine test, which excluded the main causal agents in our environment. Likewise, given that there is no method for identifying this particular toxin, the diagnosis was made based on the patient’s history of consuming the plant in question. Other diagnoses such as meningitis or encephalitis were considered, however, the patient was admitted to the intensive care unit in a hemodynamically unstable condition and with altered coagulation times, so a lumbar puncture was not considered. In addition, the patient had already received antibiotic therapy, so the cerebrospinal fluid culture could yield a false negative, taking into account that after an effective dose of antibiotics, this study could be altered. Regarding the diagnosis of Reye’s syndrome, we are referring to a patient from a rural area of Ecuador, and in reality, this population does not have access to medication such as acetylsalicylic acid, which is one of the causes. Upon questioning the family members, no pathology of any kind was reported in the patient. Various studies have demonstrated a lack of knowledge about plants among emergency department physicians. Scalise et al. conducted a study with emergency medicine doctors and nurses at an urban university hospital, in which they were asked to identify berries. None (0%) correctly identified all 10 specimens, less than 10% identified the berries correctly by their common name, and fewer still were able to correctly determine the presence or absence of potential toxicity (13). On the other hand, Harchelroad et al., in their study, asked 56 doctors to identify 12 plants. The results revealed that none (0%) correctly identified all 12 samples; 17% of the plants were correctly identified by their common name, and only 13% were correctly identified as toxic or non-toxic (14).

The information collected and available mainly focuses on the impact and immediate effects on animal health, highlighting the need and importance of further research to assess the potential risks and effects on human health.

## 7 Conclusion

Poisoning by Pascalia glauca is well-documented in animals, particularly in ruminants, where it causes severe hepatotoxicity and often fatal outcomes. This report presents the first documented case of Pascalia glauca poisoning in humans, demonstrating its potential to induce severe neurotoxic and hepatotoxic effects. The patient developed acute liver failure, metabolic encephalopathy, and coagulopathy, confirming the systemic toxicity of this plant in humans.

Given the absence of prior human cases, there is currently no established protocol for managing Pascalia glauca intoxication. This case underscores the urgent need for increased awareness among healthcare professionals regarding toxic plants and their clinical manifestations. Early recognition and timely supportive care are crucial for improving patient outcomes. Furthermore, public health efforts should focus on educating communities about the dangers of toxic plant ingestion, particularly in regions where Pascalia glauca may be inadvertently consumed. Future research should explore potential hepatoprotective interventions and establish evidence-based management strategies for similar cases.

## Data Availability

The original contributions presented in this study are included in this article/supplementary material, further inquiries can be directed to the corresponding authors.
